# Insights into lipid-protein interactions from computer simulations

**DOI:** 10.1007/s12551-021-00876-9

**Published:** 2021-11-03

**Authors:** D. P. Tieleman, B. I. Sejdiu, E. A. Cino, P. Smith, E. Barreto-Ojeda, H. M. Khan, V. Corradi

**Affiliations:** 1 Centre for Molecular Simulation and Department of Biological Sciences, University of Calgary, 2500 University Dr. NW, Calgary, AB T2N 1N4 Canada; 2 Department of Structural Biology and the Center for Data Driven Discovery, St. Jude Children’s Research Hospital, Memphis, TN USA; 3 Department of Physics, King’s College London, London, WC2R 2LS UK

**Keywords:** Lipid-protein interactions, Membrane proteins, Molecular dynamics simulations, Martini

## Abstract

Lipid-protein interactions play an important direct role in the function of many membrane proteins. We argue they are key players in membrane structure, modulate membrane proteins in more subtle ways than direct binding, and are important for understanding the mechanism of classes of hydrophobic drugs. By directly comparing membrane proteins from different families in the same, complex lipid mixture, we found a unique lipid environment for every protein. Extending this work, we identified both differences and similarities in the lipid environment of GPCRs, dependent on which family they belong to and in some cases their conformational state, with particular emphasis on the distribution of cholesterol. More recently, we have been studying modes of coupling between protein conformation and local membrane properties using model proteins. In more applied approaches, we have used similar methods to investigate specific hypotheses on interactions of lipid and lipid-like molecules with ion channels. We conclude this perspective with some considerations for future work, including a new more sophisticated coarse-grained force field (Martini 3), an interactive visual exploration framework, and opportunities to improve sampling.

## Introduction

Lipid-protein interactions have emerged as important contributions to a variety of membrane-bound processes, including the mechanism of specific membrane proteins, modulating conformational changes, larger-scale effects including the formation of membrane domains, membrane remodeling, and very likely in the overall organization of biological membranes (Brown 2017; Corradi et al. 2019; Lee 2003). Molecular dynamics (MD) simulations have reached time and length scales, including with standard atomistic models and with increasingly sophisticated larger-scale models, where they can be used for meaningful investigations of membrane systems of direct biological and biomedical interest (Marrink et al. 2019). At the same time, a rapidly growing number of experimentally determined membrane protein structures, primarily by cryo-EM (Cheng 2018; Thonghin et al. 2018), is identifying lipid or surfactant density associated with membrane proteins (Duncan et al. 2020; Sun and Gennis 2019; Thompson and Baenziger 2020), and lipidomics approaches have made a major leap in their ability to identify lipid compositions in complex membranes in general (Lorent et al. 2020; Symons et al. 2021) and, in some cases, lipids associated with membrane proteins (Bolla et al. 2019; Frick and Schmidt 2019; Sun and Gennis 2019; Teo et al. 2019; van ‘t Klooster et al. 2020). One of the long-term aims of our group is to understand the diverse roles lipid-protein interactions play in biological processes in both qualitative and quantitative terms, from a fundamental biological perspective and to use an improved understanding of lipid-protein interactions in biomedical applications. In this paper, we provide a perspective on recent work, primarily from our group, on lipid-protein interactions and reflect on future directions.

## Membrane proteins have a unique, local lipid environment

**Fig. 1:**
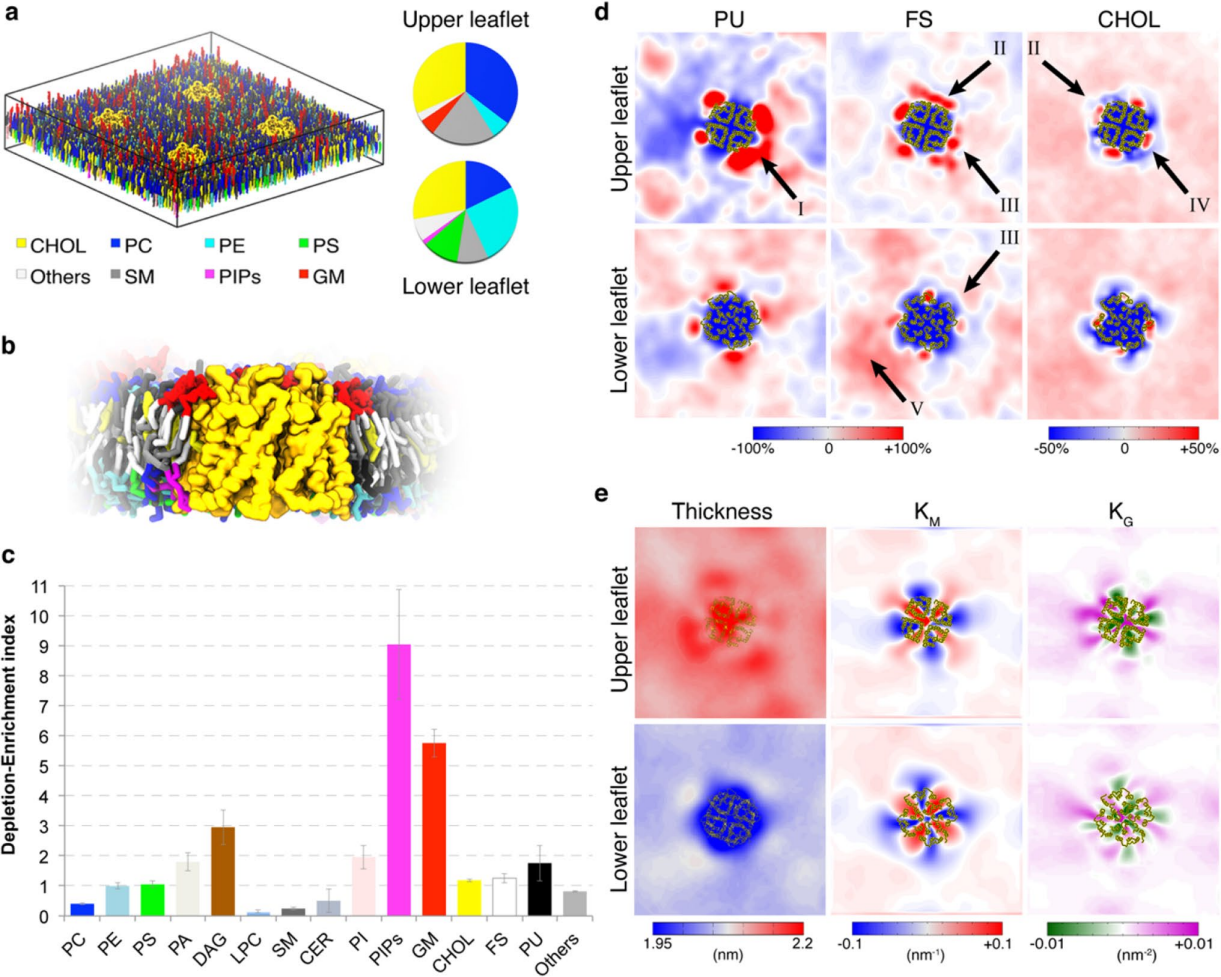
Unique lipid annular shells for Aquaporin 1 (AQP1). **a** Simulation setup for AQP1, with four copies of the protein embedded in a plasma membrane model with more than 60 lipid types. The pie charts represent the lipid head group composition of the upper and lower leaflet. **b** View of the first few lipid shells around AQP1, after 30 μs of simulation time. Lipid tails and head groups are colored as in **c**. **c** Lipid depletion-enrichment (DE) index for the AQP1 simulation, calculated from the last 5 μs of a 30-μs simulation. Shown is the average over the four AQP1 molecules. The DE index is computed by dividing the lipid composition of the annular shell by the bulk membrane composition. Values larger than 1 indicate enrichment, while values smaller than 1 indicate depletion of the chosen class (see Corradi et al. 2018 for more details on lipid classes and analyses. Membrane protein fingerprints. **d** 2D lateral density maps, showing local density fluctuations around AQP1 in upper (top row) and lower (bottom row) leaflets, grouped according to lipid classes: polyunsaturated (PU) lipids, fully saturated (FS) lipids, and cholesterol. Major observations are highlighted by arrows: I, non-specific binding; II, non-uniform distribution; III, leaflet asymmetry; IV, specific binding; V, membrane fluctuations (see Corradi et al. 2018 for more details). **e** Non-uniform variations in local membrane properties around AQP1: thickness, mean curvature, and Gaussian curvature for upper and lower leaflets. Figure panels were modified from Corradi et al. (2018)

Lipid-protein interactions have been widely recognized as key players in the function of many membrane proteins through specific interactions at well-defined binding sites (Corradi et al. 2019). They also arise by virtue of membrane proteins being in a lipid environment. Although this seems trivial, thermodynamically, and kinetically, this is a complex environment that modulates many different properties of the membrane (Enkavi et al. 2019). Lipid-protein interactions are also critical in shaping local membrane structure and may in fact be a defining organizational factor for biological membranes. Experimental techniques ranging from X-ray and electron crystallography, to fluorescence spectroscopy, cryo-electron microscopy, and mass spectrometry have provided several examples of lipids tightly bound to membrane proteins (Barrera et al. 2013; Loll 2014; Loura et al. 2010; Raunser and Walz 2009). These techniques mainly capture strong interactions, they typically are not quantitative thermodynamically, and they do not give high spatial resolution. Recently, the use of styrene-maleic acid (SMA) polymers has been applied to extract membrane proteins from their natural lipid environment, forming lipid-protein nanoparticles that can be combined with lipidomics analyses for the identification of the lipids (Barniol-Xicota and Verhelst 2021; Pollock et al. 2018; Reading et al. 2017; Teo et al. 2019; van ‘t Klooster et al. 2020). However, it remains to be clarified how well the native environment is maintained or influenced using the polymers (Barniol-Xicota and Verhelst 2021). A detailed representation of the lipid environment of membrane proteins can be achieved with computational methods such as molecular dynamics (MD) simulations, applied extensively to characterize specific and less specific lipid-protein interactions (Corey et al. 2020; Corradi et al. 2019; Enkavi et al. 2019; Muller et al. 2019). Coarse-grain (CG) models, in particular, can simulate reversible binding and unbinding events and detect strong as well as weak lipid-protein interactions (Corey et al. 2020; Hedger and Sansom 2016; Ingolfsson et al. 2014a). In addition, increasing computing power together with the development of optimized software has increased significantly the time scale, length scale, and complexity of simulated membrane systems (Marrink et al. 2019). We studied the local lipid composition of proteins representing ten eukaryotic membrane protein families (Corradi et al. 2018), embedded in a plasma membrane mixture with more than 60 lipid types (Ingolfsson et al. 2014b). Our hypothesis was that each protein will generate its own environment, distinctly different from the lipids concentrations in the bulk. Indeed, we identified unique lipid shells surrounding the proteins, and this in turn triggered non-uniform perturbations of local membrane properties, which we defined as “fingerprint.” Our results revealed a landscape of diverse lipid-protein interactions as well as protein-driven effects on membrane properties, extending the lipid-protein interplay beyond the interactions between proteins and tightly bound lipids, to include the overall structure of the membrane (Fig. 1).

## Simulations can find lipid-binding sites

In the previous section, we described simulations of a set of membrane proteins meant to sample the major eukaryotic membrane protein families, and we found significant differences for each protein. What would happen for a set of more closely related membrane proteins? Due to their essential physiology and importance as drug targets, G protein-coupled receptors (GPCRs) are among the most studied membrane proteins (Hauser et al. 2017, 2018). Initial MD simulations of rhodopsin in bilayer mixtures containing differently saturated lipids and cholesterol showed that rhodopsin interactions with lipids may affect measured stability and kinetics (Grossfield et al. 2006). Following studies, using both experimental and computational approaches, further underlined the importance of considering the lipid environment in GPCR structure and functional investigations. Results from over almost two decades of MD simulations show several different GPCRs, mainly from class A, forming consistent yet distinct interactions with cholesterol and other membrane lipids (Corradi et al. 2019; Periole 2017; Sengupta and Chattopadhyay 2015).

Because these studies use different methods, including force fields, set-up procedures, and simulation lengths, a direct quantitative comparison is challenging. To get a picture of GPCR-lipid interaction in a family-wide context, we simulated 28 different GPCR structures belonging to different classification levels and conformational states in the same complex plasma mixture described in the previous section (Sejdiu and Tieleman 2020). We found that the enrichment and depletion of selected lipid classes near the proteins show similar trends across the different GPCRs but with different magnitudes, depending on the type of receptor and on its conformational state. For instance, phosphatidylinositol-(bi, tri)phosphate lipids (PIPs) are highly enriched around the histamine H1 receptor but only modestly enriched in the case of the smoothened receptor (an interactive graphics showing data for all the studied GPCRs can be accessed through ProLint (Sejdiu and Tieleman 2021), a web application for the analysis of lipid-protein interactions, with a section dedicated to GPCRs: prolint.ca/gpcrs/stats).

**Fig. 2:**
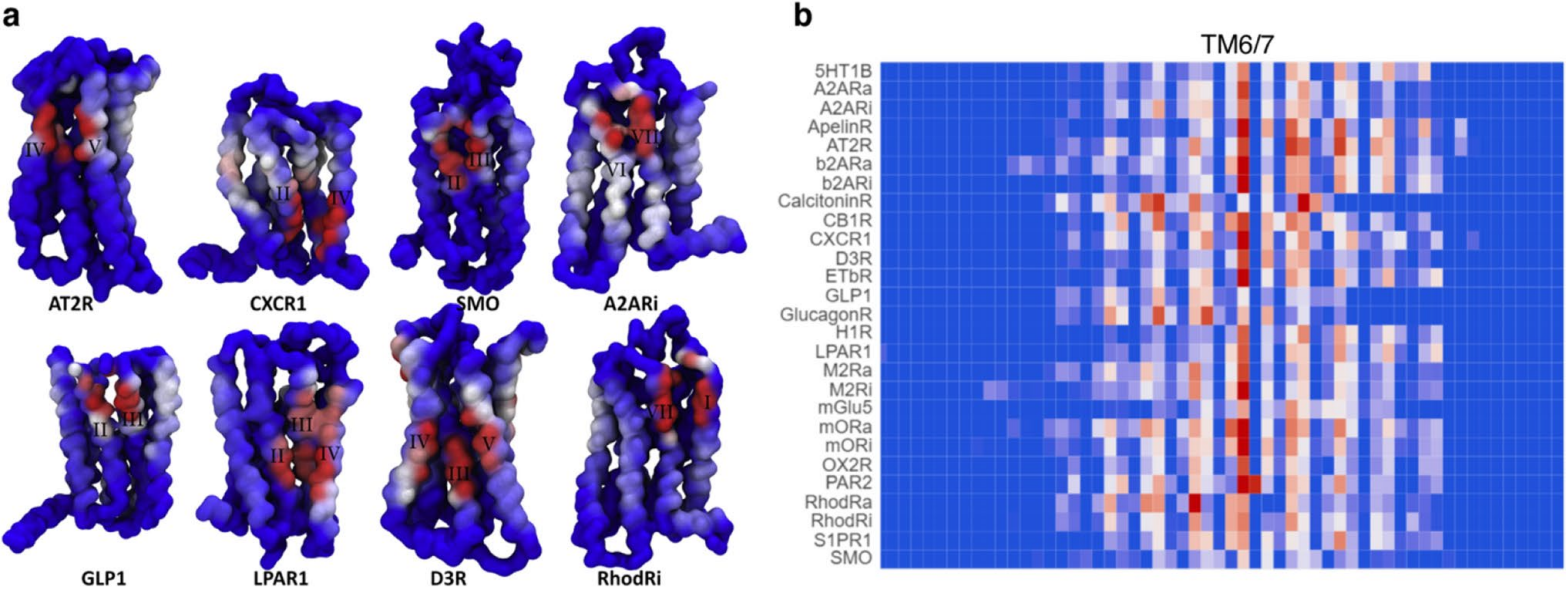
**a** GPCR-cholesterol interaction for eight GPCRs shown as a surface presentation of cholesterol contacts. Color scale (red-white-blue) represents an increase in the contact duration of cholesterol (for more details, see Sejdiu and Tieleman 2020). A larger set of GPCRs, including a detailed comparison between contact duration and contact number as visualization metrics, is given in the Supporting Information of (Sejdiu and Tieleman 2020) (figure from Sejdiu and Tieleman 2020). **b** Prevalence of GPCR-cholesterol interactions at the upper membrane leaflet-facing TM6/7 interface. Note the lack of similar interactions for the following non-class A GPCRs: calcitonin, GLP1, glucagon, and smoothened (SMO) receptors. The same color scale to highlight cholesterol interactions is used for both figures

Overall, we discovered specific interactions with both cholesterol and PIP lipids for all the simulated receptors. The number of interaction sites per structure, their interaction strength, and the location of lipid binding sites, however, differ widely between individual GPCRs. First, the binding of PIP lipids is confined to interfaces within transmembrane helices along with the intracellular loops linking them. In contrast, GPCR interactions with cholesterol (Fig. 2) require a protein environment capable of accommodating the ring structure of cholesterol and stabilizing it via hydrophobic interactions, while its head group can engage in interactions with charged residues or other lipids. Second, the large number of cholesterol interaction sites that we observed allows us to comment on the significance of CRAC/CARC motifs to cholesterol binding (Fantini et al. 2016; Jafurulla et al. 2011). While we do observe a few interactions with cholesterol that seem to be mediated by this motif, for the most part, we do not find any supporting evidence for their importance to GPCR-lipid interactions, in line with other reports (Lee 2018, 2019; Taghon et al. 2021). Third, not all interaction sites are equal. Most interactions are mediated through the extracellular side of TM2/3 and TM6-7 and the intracellular side of TM8/1. In fact, cholesterol interactions at the TM6-7 interface seem to be a common feature of class A GPCRs that is missing from their non-class A counterparts (Fig. 2; an interactive graphics showing these heatmaps is available through ProLint (Sejdiu and Tieleman 2021): prolint.ca/gpcrs/heatmap). Several crystal structures of class A GPCRs also feature a co-crystalized cholesterol at this site (Taghon et al. 2021).

## Simulations enable a molecular view of curvature partitioning consistent with experiments

Membrane curvature is a fundamental characteristic of all cells and a key aspect of the lipid raft hypothesis (Meinhardt et al. 2013; Pike 2009). Essential life processes such as cytokinesis, membrane remodeling, and vesicle formation involve curvature (Bohuszewicz et al. 2016). Some proteins show distinct curvature preferences, which suggests a connection between the local membrane environment and protein function. A prominent example is the clustering of mitochondrial ATP synthase dimers along the highly curved crista ridges and their depletion in the flat stretches of the inner membrane (Blum et al. 2019; Kuhlbrandt 2015). Still, many aspects of ATP synthase function in relation to curvature remain elusive, such as how the dimers remain anchored at specific membrane regions, and whether the curved environment influences its catalytic activity (Nirody et al. 2020). Estimates of membrane curvature free energy are well within the range required for protein conformational changes, suggesting that the elastic energy stored in the membrane may be able to allosterically regulate a vast assortment of membrane proteins (Brown 2012; Golani et al. 2019; Iversen et al. 2015). For instance, conformational changes during photoactivation of rhodopsin become more favorable as the curvature elastic energy of the membrane increases (Soubias et al. 2010), and the specific activity of diacylglycerol kinase ϵ can be over tenfold higher in curved versus flat membranes (Bozelli et al. 2018). While these observations illustrate the importance of curvature in vital biological functions, a molecular-level description is needed for a more thorough understanding of curvature-dependent processes. Due to experimental challenges associated with obtaining molecular-level data in membrane environments, MD simulations are a promising alternative. With the objective of using MD simulations to study the interplay between local membrane environment, protein shape, conformation, and function, we first investigated whether the approach yielded results consistent with experimental data.

**Fig. 3:**
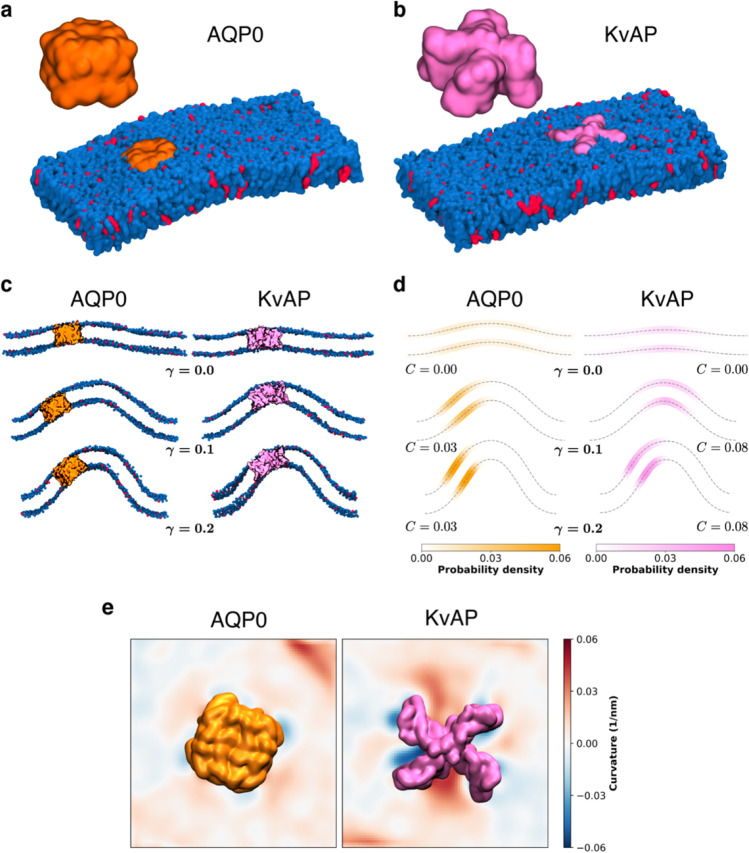
The different curvature preferences of AQP0 and KvAP are captured by Martini simulations. **a** Structure of AQP0 (orange) and snapshot of AQP0 embedded in a POPC/POPA (9:1, blue and red, respectively) membrane. **b** Structure of KvAP (pink) and snapshot of KvAP embedded in a POPC/POPA membrane. **c** Snapshots of AQP0 and KvAP in membranes of a different strain. **d** Probability density of AQP0 and KvAP in the membrane and average curvature preference, *C* (1/nm), as a function of the applied strain. **e** Spontaneous mean membrane curvature around AQP0 and KvAP in the zero strain bilayers. Each independent simulation was run for 30 μs, and the last 20 μs was analyzed in 2.5-μs blocks. Systems were built with insane (Wassenaar et al. 2015) using the Martini 2.2 parameter set (de Jong et al. 2013; Marrink et al. 2007). Minimization, equilibration, and production were carried out with GROMACS 2020 (Abraham et al. 2015) as previously described (Cino et al. 2021). Protocols for membrane buckling and data analysis followed established methods (Boyd et al. 2017) and in-house software (Barreto-Ojeda 2021)

Experimental studies from the Bassereau group revealed that the aquaporin 0 water channel (AQP0) and an archaebacterial voltage-gated potassium channel (KvAP)—two proteins of similar lateral size—have different curvature preferences in POPC/POPA (9:1 ratio) giant unilamellar vesicles (Aimon et al. 2014; Quemeneur et al. 2014). These studies highlighted how (i) AQP0 localizes to planar membrane regions, while KvAP is enriched in curved regions of the membrane; (ii) KvAP induces local membrane bending; and (iii) the lateral diffusion of KvAP is decreased in curved membranes, whereas the mobility of AQP0 is insensitive to membrane curvature. We have reproduced the experimental setup of the Bassereau group in silico by using the Martini force field (de Jong et al. 2013; Marrink et al. 2007; Marrink and Tieleman 2013) to simulate POPC/POPA/AQP0 and POPC/POPA/KvAP membranes at different degrees of membrane strain. Our CG simulations reproduce all of the aforementioned trends reported by the Bassereau group (Fig. 3), illustrating proof of principle for using the Martini force field to accurately study protein sorting in curved membranes. We thus expect that MD simulations will be a valuable tool for studying the curvature-driven modulation of protein sorting and function.

## Outlook

In eukaryotes and prokaryotes, biological membranes reveal a highly diverse lipid landscape with some lipid species found only in one or the other kingdoms of life (Sohlenkamp and Geiger 2016; van Meer et al. 2008). Eukaryotic membranes, in particular, are characterized by a lipid composition that differs at the membrane type, organelle, and leaflet level (van Meer et al. 2008; Harayama and Riezman 2018). The asymmetric distribution of lipids between leaflets and laterally within the same leaflet (Lorent et al. 2020; Symons et al. 2021; van Meer et al. 2008) is important for mechanical properties as well as for many cellular functions, including but not limited to protein recruitment and protein function regulation, signaling, and energy storage (Lorent et al. 2020). There are also crucial mechanistic connections between membranes and the cytoskeleton, linked to the presence of specific lipid types and domains (Bezanilla et al. 2015; Head et al. 2014).

Molecular-level details of vital processes that take place in the membranes can now be investigated using molecular dynamics (MD) simulations, and recent reviews provide a comprehensive overview of the challenges in this field and the mechanistic details of membrane proteins and their interplay with lipids that can be revealed with simulations (Corradi et al. 2019; Marrink et al. 2019; Muller et al. 2019). Here we have highlighted three applications of MD simulations to specific biophysical questions based on lipid-protein interactions. The first one shows that relatively small changes in local lipid concentration around a given protein result in a unique environment for each protein, with biophysical properties that differ from a bulk lipid environment and may give rise to long-range organizational effects in the membrane as a whole. Our study of GPCRs shows that the lipid environment around evolutionarily related proteins is on the one hand less different than the wide range of proteins in the previous section but still shows significant differences. This work also showed that families of closely related GPCRs are more similar in their environment than more distant relatives and shows that the local lipid environment can be conformation dependent. The third example hints at a more dramatic conformation-dependent effect. We showed that two proteins of different shape partition into areas of different curvature. Thus, proteins modify the local curvature of a membrane but this also provides a driving force for partitioning to specific areas. All three examples are at a relatively global level, although the GPCRs also show clear binding sites for cholesterol and PIP lipids. In other recent work, we targeted channels that play a role in cardiac arrhythmias and we identified more specific binding sites for lipid and lipid-like ligands, including ceramides that bind and modulate the HERG potassium channel (Miranda et al. 2021) and poly-unsaturated fatty acid derivatives that modulate the KCNQ1 potassium channel (Yazdi et al. 2021).

There are a number of areas where technical progress would be desirable. The studies we highlighted all use the Martini force field, a commonly used coarse-grained force field based on a four-to-one mapping scheme, where each particle represents approximately four heavy atoms. This design of the force field provides a smoother energy surface with a reduced number of degrees of freedom, thus allowing to reach time scales of tens to hundreds of microseconds (de Jong et al. 2013; Marrink et al. 2007; Marrink and Tieleman 2013). However, sampling is a major concern in atomistic and coarse-grained simulations in general, but specifically for lipid-protein interactions, the time scale is limited by the diffusion rates of lipids. A pipeline that incorporates atomistic simulations based on previous Martini sampling is a compromise to obtain more detail, but even with the special purpose Anton supercomputer for molecular dynamics simulations (Shaw et al. 2014), atomistic simulations are limited typically to microseconds. Although computers will no doubt continue to become faster, there is room for improvement in both sampling and coarse-grain models. We recently showed that for Martini simulations of lipid mixtures, it is possible to greatly increase sampling by randomly swapping lipids across the system rather than waiting for them to diffuse (Cherniavskyi et al. 2020). Although an extension to protein systems is not straightforward, this seems worthwhile to pursue. Martini has been widely adopted to simulate lipid-protein systems, with most papers using Martini 2. There are limitations to this approach (Alessandri et al. 2019; Corradi et al. 2019; Marrink et al. 2019; Muller et al. 2019). Recently, a major update of the force field has enabled mixed resolutions (Souza et al. 2021), and a much more accurate description of small molecules (Souza et al. 2020) and further development is ongoing on improved descriptions of lipids and proteins. Martini 3 retains the speed advantage of Martini but promises additional accuracy in detailed interactions as a further step between coarse-grain simulations and detailed molecular interactions.

MD simulations clearly can give very detailed information about lipid-protein interactions. One challenge is to improve the crosstalk with experimental studies. In part, this is due to the importance of simulating more realistic membrane compositions for longer time scales, but also the technical challenge in accurately identifying specifically bound lipids and the challenge in sharing simulation results. The first two sections above describe dozens of simulations, but analysis in published papers is limited by space and imagination, although there is much more data available in the raw simulation files. To simplify the analysis of lipid-protein interactions and system-independent visualization of results, including results not highlighted in papers, we have developed ProLint, an open-source webserver and set of tools that completely automates analysis and visualization of lipid-protein interactions (Sejdiu and Tieleman 2021). The visualization application linked (Fig. 2) above are part of ProLint (www.prolint.ca) and they highlight its capability in making simulation results available to the entire scientific community. We hope that ProLint’s ability to visualize lipid-protein interactions using different web-based applications will make MD-generated data more accessible to experimentalists and encourage discussions, motivate collaborations, and accelerate future research in lipid-protein interactions.

## Data Availability

Not applicable.
